# Mitochondrial genome mutations and neuronal dysfunction of induced pluripotent stem cells derived from patients with Alzheimer's disease

**DOI:** 10.1111/cpr.13274

**Published:** 2022-06-13

**Authors:** Yeonmi Lee, Minchul Kim, Miju Lee, Seongjun So, Soon‐Suk Kang, Jiwan Choi, Deokhoon Kim, Hyohoon Heo, Sung Soo Lee, Hee Ra Park, Jung Jae Ko, Jihwan Song, Eunju Kang

**Affiliations:** ^1^ Department of Biomedical Science CHA University Seongnam Gyeonggi‐do Republic of Korea; ^2^ Center for Embryo & Stem Cell Research CHA Advanced Research Institute Seongnam Gyeonggi‐do Republic of Korea; ^3^ iPS Bio, Inc. Seongnam Republic of Korea; ^4^ Department of Pathology, Asan Medical Center University of Ulsan College of Medicine Seoul Republic of Korea

## Abstract

**Objectives:**

Patient‐derived induced pluripotent stem cells (iPSCs) are materials that can be used for autologous stem cell therapy. We screened mtDNA mutations in iPSCs and iPSC‐derived neuronal cells from patients with Alzheimer's disease (AD). Also, we investigated whether the mutations could affect mitochondrial function and deposition of β‐amyloid (Aβ) in differentiated neuronal cells.

**Materials and Methods:**

mtDNA mutations were measured and compared among iPSCs and iPSC‐derived neuronal cells. The selected iPSCs carrying mtDNA mutations were subcloned, and then their growth rate and neuronal differentiation pattern were analyzed. The differentiated cells were measured for mitochondrial respiration and membrane potential, as well as deposition of Aβ.

**Results:**

Most iPSCs from subjects with AD harbored ≥1 mtDNA mutations, and the number of mutations was significantly higher than that from umbilical cord blood. About 35% and 40% of mutations in iPSCs were shared with isogenic iPSCs and their differentiated neuronal precursor cells, respectively, with similar or different heteroplasmy. Furthermore, the mutations in clonal iPSCs were stable during extended culture and neuronal differentiation. Finally, mtDNA mutations could induce a growth advantage with higher viability and proliferation, lower mitochondrial respiration and membrane potential, as well as increased Aβ deposition.

**Conclusion:**

This study demonstrates that mtDNA mutations in patients with AD could lead to mitochondrial dysfunction and accelerated Aβ deposition. Therefore, early screening for mtDNA mutations in iPSC lines would be essential for developing autologous cell therapy or drug screening for patients with AD.

## Graphical abstract

mtDNA mutations were found in induced pluripotent stem cells derived from patients with Alzheimer's disease. The mutations could induce growth advantage due to their high viability and proliferation. Differentiated neuronal cells with mtDNA mutations exhibited mitochondrial and neuronal dysfunction, as well as increased Aβ deposition.

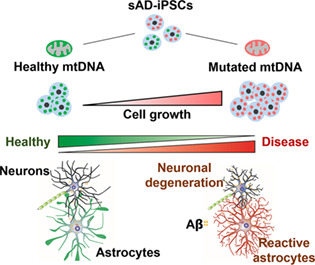

## 1 INTRODUCTION

Autologous stem cell therapy using patient‐derived induced pluripotent stem cells (iPSCs) is a promising approach, which can cause fewer concerns about immune rejection and ethical controversies.^1^, ^2^, ^3^ Patient‐derived iPSCs also offer an innovative new tool for disease modeling and drug screening as they can recapitulate physiological and biochemical properties of the disease.^4^, ^5^, ^6^

Alzheimer's disease (AD) is the most common, age‐related neurodegenerative disease. The late‐onset sporadic AD (sAD) is the major form, in which a complex combination of genetic and epigenetic changes, environmental factors, and lifestyle influences the incidence of sAD.^7^ The familial form of AD (fAD) is predominantly early‐onset and caused by specific genetic defects, which can be inherited.^7^ In both cases, mitochondrial dysfunction has been implicated in the symptoms of the disease,^8^ and several reports demonstrated an increased ratio of mtDNA mutation in the brain of patients with AD.^9^, ^10^ However, it has yet to be elucidated whether the mutations detected in patients with AD can affect the cellular function and phenotype.

The elderly derived iPSCs showed serious disadvantages in mitochondrial integrity when compared with other stem cells derived from fetal or embryonic sources.^11^, ^12^ Especially, iPSCs with mtDNA mutations could lead to cellular respiratory defects.^12^, ^13^ However, mtDNA mutations in iPSCs were not often detected in parental cells because they could occur randomly in individual cells. Therefore, it is necessary to investigate mtDNA mutations in single cell‐derived iPSC from patients and evaluate the pathogenesis of their specific mtDNA mutations.

Taken together, we analyzed mtDNA mutations in iPSCs derived from patients with AD and elderly subjects and evaluated the dynamics of mtDNA mutations during expended culture. We also differentiated iPSCs into the neuronal lineage and explored how mtDNA mutation could induce mitochondrial dysfunction and pathogenesis in differentiated cells.

## 2 MATERIALS AND METHODS

### 2.1 Ethics

All experiments were performed according to appropriate guidelines and regulations. The use of human material was approved by the Institutional Review Board of CHA University (104308‐201,612‐BR‐031‐09) and informed consent was obtained from the participants.

### 2.2 iPSCs generation from mononuclear cells

iPSCs generation was performed as previously described.^14^ Briefly, mononuclear cells (MNCs) were isolated from the peripheral blood of the subjects using the Ficoll‐Paque PLUS method (GE Healthcare). Isolated MNCs were cultured in a 24‐well plate for 4 days in MNC media: StemFit Basic02 medium (Ajinomoto) containing 50 ng/ml interleukin‐6, 50 ng/ml stem cell factor, 10 ng/ml thrombopoietin, 20 ng/ml Flt3 ligand, 20 ng/ml interleukin‐3, and 10 ng/ml granulocyte colony‐stimulating factor (all from WAKO). For induction of iPSCs, 1 × 10^5^ MNCs were transferred into six‐well dishes containing MNC media with SeVdp (KOSM) 302L (a gift of Dr Mahito Nakanishi, Tokiwa‐Bio Inc.) at MOI of 3, coated with iMatrix‐511 Silk (Matrixome). On the next day, 500 μl of StemFit Basic02 medium containing 100 ng/ml of basic fibroblast growth factor (bFGF) (Peprotech) was added every day for 4 days. The medium was completely replaced every other day from Day 5 until iPSC‐like colonies appeared.

### 2.3 iPSC culture

All iPSC lines were maintained in Stemfit Basic02 medium containing 100 μg/ml bFGF (100‐18B; Peprotech) on the iMatirx‐511 Silk‐coated dish. A tissue culture dish was coated with 0.5 μg/cm^2^ iMatrix‐511 Silk, diluted in phosphate‐buffered saline (PBS) at room temperature (RT) overnight. On Day 7, fully frown iPSCs were washed with PBS, treated with 1 ml 0.5X TrypLE solution (12563‐011; Gibco), and incubated in a 37°C, 5% CO_2_ incubator for 5 min. The 0.5X TrypLE solution consisted of a 1:1 solution of 1X TrypLE Select and a 0.5‐μM EDTA (Sigma) in PBS. After incubation, the 0.5X TrypLE solution was removed, and 1 ml Stemfit Basic02 medium containing 10 μM Y‐27632 (1293823; Peprotech) was added. These cells were then separated using a cell scraper (90020; SPL Life Sciences). The separated cells were collected in a 15 ml conical tube and their cell counts were measured using trypan blue staining. Approximately 1 × 10^4^ cells were resuspended in the Stemfit Basic02 medium containing 10 μM Y‐27632 and were seeded on the precoated dish. After 24 h, the medium was replaced with a fresh Stemfit Basic02 medium without Y‐27632. The medium was changed the next day, afterwards, the medium was changed every other day.

### 2.4 mtDNA sequencing by MiSeq

The mtDNA sequencing was performed as previously described.^12^ The whole mitochondrial genome was amplified by dividing it into two pairs using the following primers: F: mt7272‐GGCTCATTCATTTCTCTAACAGC and R: mt15712‐TTGGCTTAGTGGGCGAAATA; F: mt15635‐TCCATCCTCATCCTAGCAAT and R: mt7401‐GGGGGCATCCATATAGTCAC. PCR conditions were 98°C for 30 s, then 35 cycles of 98°C for 10 s, 56°C for 10 s, and 72°C for 4 min; 35 cycles of 72°C for 5 min. The concentrations of PCR products were measured using the Qubit 2.0 Fluorometer (Invitrogen). The amplified DNA was used for library preparation with the Nextera XT DNA Kit (Illumina). Sequencing was also performed on the Illumina MiSeq platform (Asan Medical Center), and the data were analyzed using the NextGENe software. The sequence reads in the range of 100–200 bp were quality filtered and processed using the NextGENe software and BLAT. The sequence error correction (i.e., condensation) was performed to reduce false‐positive and to generate sample consensus sequence and variant calls. Alignment with no sequence condensation was used to evaluate the percentage of the mitochondrial genome with a coverage depth of 1000. Based on the quality FASTQ reads, the read quality was filtered and converted to FASTA format. The filtered reads were aligned to the human mtDNA reference NC_012920.1, and then a variant call was executed. The heteroplasmy of the variant was calculated using the NextGENe as follows: base heteroplasmy (mutant allele frequency %) = mutant allele (forward + reverse)/total coverage of all alleles C, G, T, and A (forward + reverse) × 100.

### 2.5 Sanger sequencing

The mt3243T>C and the mt14319T>C mutations were sequenced using the following primer sets. For mt3243T>C; mt2941F‐GCGCAATCCTATTCTAGAGT and mt4632R‐CTTCTGTGGAACGAGGGTTT; for mt14319T>C; mt13837F‐GCCCTAGACCTCAACTACCT and mt14570R‐GCGGTGTGGTCGGGTGTGTT were used. The PCR reaction was performed using the 2X PCRBIO HS Taq Mix Red Kit (PCR Biosystems). Sequencing was conducted by Macrogen, and the data were analyzed using SnapGene v.5.2.

### 2.6 Cell viability assay

All experiments to evaluate cell viability were performed, as described previously.^15^ To evaluate cell growth, iPSCs were seeded at 1 × 10^5^ on a six‐well plate, and after 6 days, the cells were dissociated into single cells using 0.25% trypsin–EDTA, and the number was counted. The fold increase in cell number was used to determine cell growth. The apoptotic analysis was performed using the Dead Cell Apoptosis Kit with Annexin V Alexa Fluor 488 and propidium iodide (PI) (Invitrogen). iPSCs were seeded at 1 × 10^6^ on a six‐well plate, and after 1 day, the floating and attached cells were collected and stained with Annexin V/PI following the manufacturer's instruction. Stained cells were further analyzed using FACS (Canto II; BD Biosciences). iPSCs were seeded at 2 × 10^5^ on a six‐well plate and incubated for 2 days to determine proliferation. Next, the cells were collected and fixed with 10% neutral buffered formalin (BBC Biochemical) for 30 min at RT. The fixed cells were washed twice with PBS (Hyclone) and permeabilized with 0.05% Triton X‐100 in 0.01 M sodium citrate for 30 min at RT. Antibody against Ki‐67 (Abcam) was diluted at 1:200 in PBS containing 10% fetal bovine serum (Life Technologies) and incubated with cells overnight at 4°C. The cells were washed three times with PBS and incubated for 1 h at 4°C with goat anti‐rabbit IgG H&L (1:500; Alexa Fluor 555; Abcam). The Ki‐67‐positive cells were counted using FACS (Canto II; BD Biosciences), and the data were analyzed with FlowJo x v.0.7 software (Tree Star).

### 2.7 Differentiation into neuronal precursor cells

Human iPSCs were dissociated into single cells using 0.5% TrypLE Select, and the single cells were reaggregated into embryoid bodies (EBs) in a 96‐well Nunclon Sphera Microplate (Thermo Fisher Scientific). The EBs were cultured in Dulbecco's modified Eagle's medium (DMEM)/F12 containing 20% knockout serum replacement, 0.1 M nonessential amino acids (NEAA), 0.1 M 2‐mercaptoethanol, 0.1% antibiotic–antimycotic solutions (all from Thermo Fisher Scientific), 10 μM SB431542 (Reagent Direct), and 10 μM LDN 193189 (Sigma) for neuronal induction for 8 days. After neuronal induction, they were dissociated using Accutase (Stem Cell Technologies) and were seeded onto 15 μg/ml poly‐l‐ornithine (Sigma) and 5 μg/ml laminin (Sigma)‐coated dishes. Neuronal precursor cells (NPCs) were cultured in DMEM/F12 media containing 0.1 M NEAA, 0.1 M 2‐mercaptoethanol, 0.1 antibiotic–antimycotic solutions, 1% d‐glucose (Life Technologies), 1% B27 supplement without vitamin A (Thermo Fisher Scientific), 200 mM l‐glutamine (Sigma), and 40 ng/ml bFGF (PeproTech) for further passaging.

### 2.8 Differentiation into cortical neurons

NPCs were differentiated into cortical neuronal cells for 16 weeks in Neurobasal A medium containing 2% B27 supplement, 1% N2 supplement, 1X GlutaMAX (all from Thermo Fisher Scientific), 200 nM l‐ascorbic acid, 20 ng/ml brain‐derived neurotrophic factor, 20 ng/ml glial cell‐derived neurotrophic factor, and 20 ng/ml neurotrophin‐3 (all from PeproTech) on PLO/laminin‐coated dishes. All media were supplemented with a 1% antibiotic–antimycotic solution (Thermo Fisher Scientific). In addition, 1 × 10^4^ cells/well were seeded on a 96‐well plate (Greiner), and the half volume of media was changed in subsequent days.

### 2.9 Assessment of mitochondrial respiratory function

Mitochondrial respiratory rate was assessed using the XF Cell Mito Stress Test Kit in the XF24 Extracellular Flux Analyser (Seahorse Biosciences), as previously described.^12^ Mitochondrial oxygen consumption rate (OCR) was measured by the serial addition of oligomycin (2 μM) for ATP production (oligomycin OCR–basal OCR), carbonyl cyanide 4‐(trifluoromethoxy) phenylhydrazone (1 μM) for maximal respiration and spare respiratory capacity (maximal OCR–basal OCR), and antimycin A (0.5 μM) and rotenone (0.5 μM) for nonmitochondrial oxygen usage. The value was normalized to the baseline oxygen consumption with 1 ng of DNA.

### 2.10 Immunocytochemistry

Samples were fixed with 4% paraformaldehyde (Biosesang) overnight at 4°C. The fixed samples were permeabilized and washed with TPBS containing 0.2% Triton X‐100 (Sigma) and 0.01% Tween‐20 (Biosesang) in PBS blocked with 3% normal horse serum (Vector Labs) in TPBS for 2 h at RT. The following primary antibodies were prepared in blocking solution for incubation overnight with gentle rocking at 4°C: anti‐Nestin (1:200; R&D Systems), anti‐MAP2 (1:200; Thermo Fisher Scientific), anti‐S100β (1:200; Abcam), anti‐GFAP (1:200; DAKO), anti‐β‐amyloid (anti‐Aβ), 1–16 antibody (clone 6E10) (1:100; Biolegend), anti‐LC3B (1:100; Cell Signaling), and anti‐human mitochondria (anti‐hMito) (1:100; Abcam). Afterward, the cells were washed with TPBS three times and were incubated with secondary antibodies (Thermo Fisher Scientific) for 2 h at RT with gentle rocking, and then counterstained with DAPI for 30 min in PBS after additional three washes with TPBS. The fluorescence images were acquired using the ImageXpress Micro Confocal (IXMC) microscopy for high content analysis with the Metaxpress6 program v.6.6.3.55 (Molecular Devices).

### 2.11 Mitochondrial membrane potential

The live cells were treated with 5 μg/ml JC‐1 dye (Thermo Fisher Scientific) for 30 min in a 37°C incubator with 5% CO_2_ exposure. After incubation, the cells were washed three times with DPBS and fixed with 4% paraformaldehyde overnight at 4°C. Before imaging samples in the IXMC microscope, fixatives were removed and the samples were washed three times with DPBS. Ninety‐nine images from cortical neuronal samples were obtained at ×60 magnification with 10 *z*‐planes of 3‐μm slices. The two‐dimensional (2D) projection images were quantified for the integrated intensity of JC‐1 dyes emitted at 530 and 590 nm wavelengths using the MetaXpress software. The Find Blobs module was used to identify each JC‐1‐positive object 0.5–2 μm in size >500 grey levels above the local background. In contrast, the Filter Mask module excluded the nonspecific signals >5000 grey levels. The ratio between the sum of the total integrated intensity of JC‐1 red and green was processed and visualized using Excel. The same methods were applied to NPCs, and 36 images for each sample were obtained at ×20 magnification with three *z*‐planes of 3‐μm slices.

### 2.12 Mitophagy analysis

The fixed cells were stained with anti‐hMito and anti‐LC3B to capture the mitochondria colocalized with anti‐LC3B‐positive autophagosome. Thirty‐six images were obtained at ×20 magnification using IXMC microscopy with 10 *z*‐planes of 3‐μm slices. The best focused 2D projection images were analyzed using the Find Blobs of MetaXpress software, to identify anti‐LC3B‐positive objects 1–5 μm in size >100 grey levels above the local background, and anti‐hMito‐positive objects 0.1–3 μm in size >500 grey levels above the local background. Filter Mask program was used to exclude nonspecific signals from anti‐LC3B‐positive signals ˃500 grey levels and anti‐hMito‐positive signals ˃4000 grey levels above the background, and the logical operation “AND” was used to generate the colocalized population between LC3B and hMito masks from previous modules. Finally, the ratio of mitochondria colocalized with autophagosome from the total number of mitochondria for each image was processed and visualized using Excel.

### 2.13 Populations and outgrowth of differentiated cells

The fixed cells were stained with anti‐MAP2 for neuronal populations and anti‐S100β for glial cell populations. All 36 images for each sample were taken using IXMC microscopy at ×20 magnification with 10 *z*‐planes of 3‐μm slices. The 2D projection images were analyzed using the outgrowth program of the MetaXpress software to identify MAP2‐positive neurite outgrowth from cell bodies with 30 μm for the maximum width and 80 μm^2^ for the minimum area >2000 grey levels above the local background. Also, the neurites projected from cell bodies were identified using the parameters of 10 μm for the maximum width and >1000 grey levels above the local background. The same method was applied to identify s100β‐positive astrocytic outgrowth from cell bodies using the parameters of 30 μm for the maximum width, 100 μm^2^ for the minimum area, and >2000 grey levels above the local background. Also, astrocytic branches projected from cell bodies were identified using the parameters of 10 μm for the maximum width and >500 grey levels above the local background. The raw data regarding the total number, the length, and the number of branches or branching points of MAP2 and S100β‐positive cells were further processed and analyzed using Excel.

### 2.14 Accumulation of Aβ in differentiated neuronal cells

The samples were stained using an anti‐Aβ antibody paired with anti‐MAP2 for neurons or anti‐GFAP for astrocytes. Thirty‐six images for each ICC sample were taken by IXMC microscopy at ×20 magnification with 10 *z*‐planes of 3‐μm slices. The best focused 2D projection images were analyzed by the Find Blobs modules provided from MetaXpress software to identify Aβ objects 1–3 μm in size >500 grey levels above the local background. Also, adaptive threshold modules were used to identify anti‐MAP2‐positive neuronal cells 1–25 μm in size >2000 grey levels and anti‐GFAP‐positive glial cells 2–40 μm in size >3000 grey levels above the local background. Most importantly, the logical operation module “AND” was used for grouping anti‐Aβ‐positive signals within the stained area of each cortical neuronal cell marker. The raw data, including the sum of total counts, stained areas, and the integrated intensity of all masks generated by quantitative image analysis, were further processed and analyzed using Excel. The representative images were obtained using confocal microscopy (ZEISS LSM880).

### 2.15 Statistical analysis

Statistical comparisons between two groups were made using the Student's two‐tailed *t*‐test. In addition, a one‐way analysis of variance with Tukey post hoc analysis was applied when more than two groups were compared. Statistical analyses were performed using Prism 8.0.1 software (GraphPad). Data were reported as mean ± SD or SEM.

## 3 RESULTS

### 3.1 mtDNA mutations in iPSCs derived from patients with AD

**FIGURE 1:**
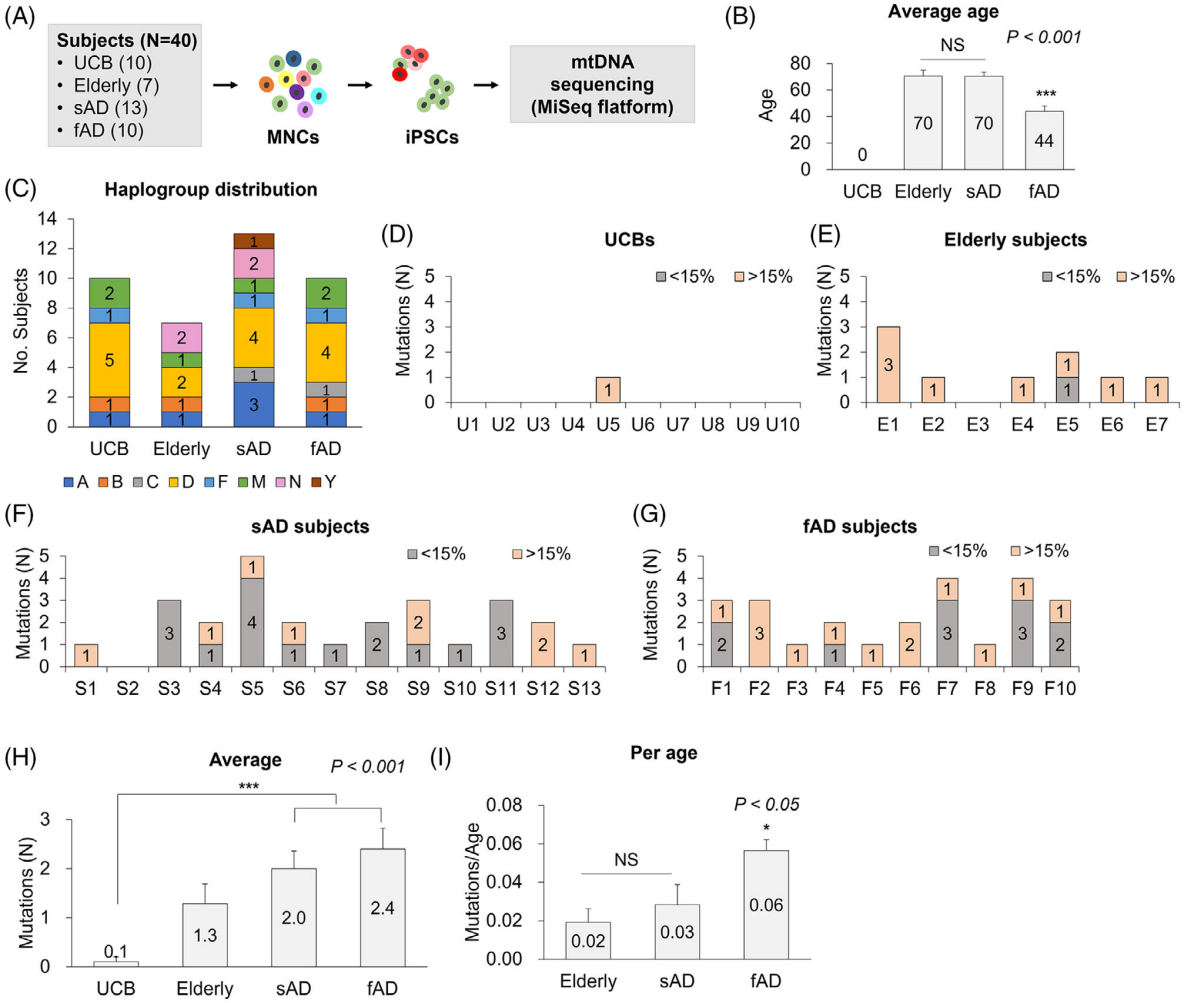
mtDNA mutations in iPSCs derived from Alzheimer's patients. (A) The scheme for mtDNA sequencing in iPSCs. Mononuclear cells (MNCs)‐derived iPSC lines from 10 umbilical cord blood (UCB), 7 elderly individuals without Alzheimer's disease (AD) symptoms, 13 patients with sporadic AD (sAD), and 10 patients with familial AD (fAD) were sequenced for mtDNA using MiSeq platform. (B) The average ages of the elderly and sAD subjects were significantly higher than those of fAD subjects. ***p < 0.001. (C) The distribution of mtDNA haplogroups. Eight haplogroups were observed in 40 subjects, which showed no association with a particular subject group. (D–G) The numbers of mtDNA mutations in iPSC lines derived from UCB (D), elderly (E), sAD (F), and fAD (G). One iPSC line (iPSC1) was analyzed for each subject. (H) Average numbers of mtDNA mutations in each group. The iPSCs of the UCB group showed a significantly lower number of mtDNA mutations than those from the sAD and fAD groups. ***p < 0.001. (I) The number of mtDNA mutations was divided by the age of subjects, in which the fAD group showed a significantly higher number than the elderly and sAD group. *P < 0.05. Mean ± SEM. iPSC, induced pluripotent stem cell; NS, not significant

mtDNA mutations were analyzed in iPSC lines derived from MNCs; 10 umbilical cord blood (UCB), 7 elderly individuals without AD symptoms, 13 patients with sAD, and 10 patients with fAD (Figure 1A and Table S1). The average ages of the elderly and sAD subjects were similar (70 years old), whereas the fAD subjects were younger (44 years old) due to the senescence‐independent onset (Figure 1B).^7^ We observed eight different mtDNA haplogroups in 40 subjects. The individual mtDNA haplogroups showed no association with a particular subject group (Figure 1C).

First, we analyzed mtDNA mutations in one iPSC line (iPSC1) for each subject (Tables [Link], [Link]). The mutations were counted in coding regions such as protein, rRNA, and tRNA encoded. Only one mutation was observed in one iPSC line out of 10 iPSC lines derived from UCB (Figure 1D), whereas nine mutations were observed in six iPSC lines among seven elderly subjects (Figure 1E). Among 13 patients with sAD, 26 mutations were observed in 12 iPSC lines (Figure 1F), and 23 mutations were detected in 10 iPSC lines of patients with fAD (Figure 1G). The iPSCs from UCB carried a significantly lower number of mtDNA mutations than those from patients with sAD and fAD (Figure 1H). The number of mtDNA mutations in patients with AD was significantly higher than in UCB but slightly higher than in the elderly of similar age, but not significant. When the number of mutations was divided by the age of subjects, the mutation rate per age in the patients with fAD was significantly higher (two to three folds) than in the elderly and sAD groups (Figure 1I), suggesting the presence of certain genetic factors that could trigger fAD lead to mtDNA instability.

In this study, the mtDNA mutation‐free iPSC line was rarely detected in the elderly and subjects with AD‐derived cells; only 1 out of the 7 elderly subjects, 1 out of the 13 patients with sAD, and none of the 10 patients with fAD (Figure 1E–G). These results demonstrate that mitochondrial integrity cannot be guaranteed for the elderly derived, particularly disease‐derived iPSCs. Therefore, screening of mtDNA mutation‐free iPSCs would be crucial to ensure the safety of future applications.

### 3.2 Comparison of mtDNA mutations in MNCs and their isogenic iPSC lines

**FIGURE 2:**
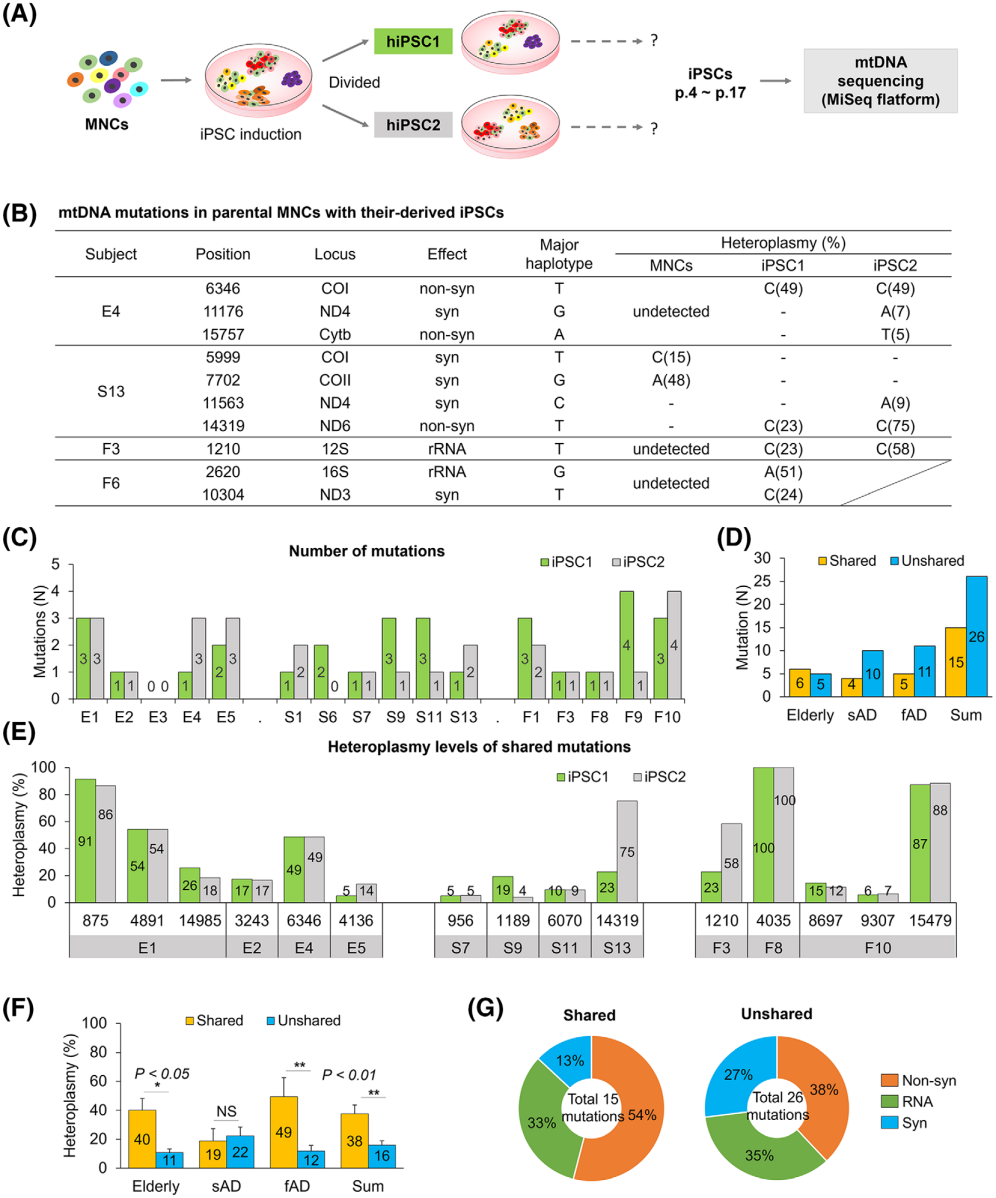
Comparison of mtDNA mutations of iPSC lines from the identical subjects. (A) The scheme of mtDNA sequencing in two iPSC lines that were derived from individual subjects. mtDNA mutations were analyzed in two iPSC lines of the identical subject using the MiSeq platform. (B) mtDNA mutations in parental MNCs with their derived iPSCs. (C) The number of mtDNA mutations in each iPSC line of five elderly, six sAD, and five fAD subjects. (D) The number of shared and unshared mtDNA mutations in subject groups. Fifteen mutations were detected in both iPSC lines, and the remaining 26 mutations were detected only in one iPSC line. (E) The heteroplasmy of shared mtDNA mutation in iPSCs. Most of the shared mutations showed a similar heteroplasmy between iPSC1 and iPSC2. (F) The average heteroplasmy of mtDNA mutations in subject groups. In the total subjects, shared mutations showed significantly higher heteroplasmy than unshared mutations. Mean ± SEM. *p < 0.05; **p < 0.01. (G) The distribution of mutation types for shared and unshared mutations. fAD, familial Alzheimer's disease; iPSC, induced pluripotent stem cell; MNC, mononuclear cell; non‐syn, nonsynonymous; syn, synonymous; RNA, tRNA, or rRNA genes; sAD, sporadic Alzheimer's disease

When there were additional iPSC lines in the same subject, mtDNA mutations in MNCs and two iPSC lines of each subject were compared (Figure 2A). First, only four parental MNCs (E4, S13, F3, and F6) and their derived iPSCs were compared to mtDNA mutations due to the limitation of MNCs samples (Figure 2B). The MNCs from E4, F3, and F6 subjects showed no mutations, while their iPSCs carried six unique mutations. Two of them were shared between two iPSC lines. The S13 subject showed two mutations in MNCs, 15% at mt5999 and 48% at mt7702. These two mutations were not found in two iPSC lines, but new two mutations were identified, 9% (iPSC2) at mt11563 and 23% (iPSC1) and 75% (iPSC2) at mt14319.

These phenomena are similar to the previous observations, in which random and novel mtDNA mutations in iPSCs take place, and the mutations occur through positive or negative selection during iPSC induction or culture.^12^ Furthermore, the mutations in iPSCs originating from somatic cells, could not be detected in pooled parental cells due to the large number of individual cells containing unique mutations.

More than two iPSC lines were generated in five elderly (E1–E5), six sAD (S1, S6, S7, S9, S11, and S13), and five fAD subjects (F1, F3, F8, F9, and F10). Among them, none to four mtDNA mutations were detected in each iPSC line (Figure 2C and Tables [Link], [Link]). We investigated whether these mutations were shared between two iPSC lines. Among 41 mutations, 15 mutations were detected in both iPSC lines (6 for elderly, 4 for sAD, and 5 for fAD subjects), and the remaining 26 mutations were detected only in one iPSC line (5 for elderly, 10 for sAD, and 11 for fAD subjects) (Figure 2D). Most of the shared mutations displayed a similar level of heteroplasmy between iPSC1 and iPSC2, whereas some mutations showed differences (Figure 2E).

When the average heteroplasmy of shared and unshared mutation was compared, the heteroplasmy of elderly and fAD subjects was significantly higher in shared mutations, whereas sAD displayed a similar level (Figure 2F). Furthermore, the shared mutations showed significantly higher heteroplasmy among the total subjects, compared with unshared mutations (Figure 2F). For example, among 15 shared mtDNA mutations, 54% were nonsynonymous substitutions in coding regions, resulting in amino acid changes, and 33% were found in tRNA or rRNA genes (Figure 2G). However, only 38% were nonsynonymous substitutions in the unshared mutation (Figure 2G), whereas 35% of the mutations were located in the RNA region, similar to shared mutations.

In summary, the shared mtDNA mutations detected in two isogenic iPSC lines showed a significantly higher heteroplasmy than in the unshared mutation. In addition, the nonsynonymous substitutions were detected slightly more in the shared mutations than in the unshared mutations.

### 3.3 mtDNA mutations in NPCs derived from AD iPSCs

**FIGURE 3:**
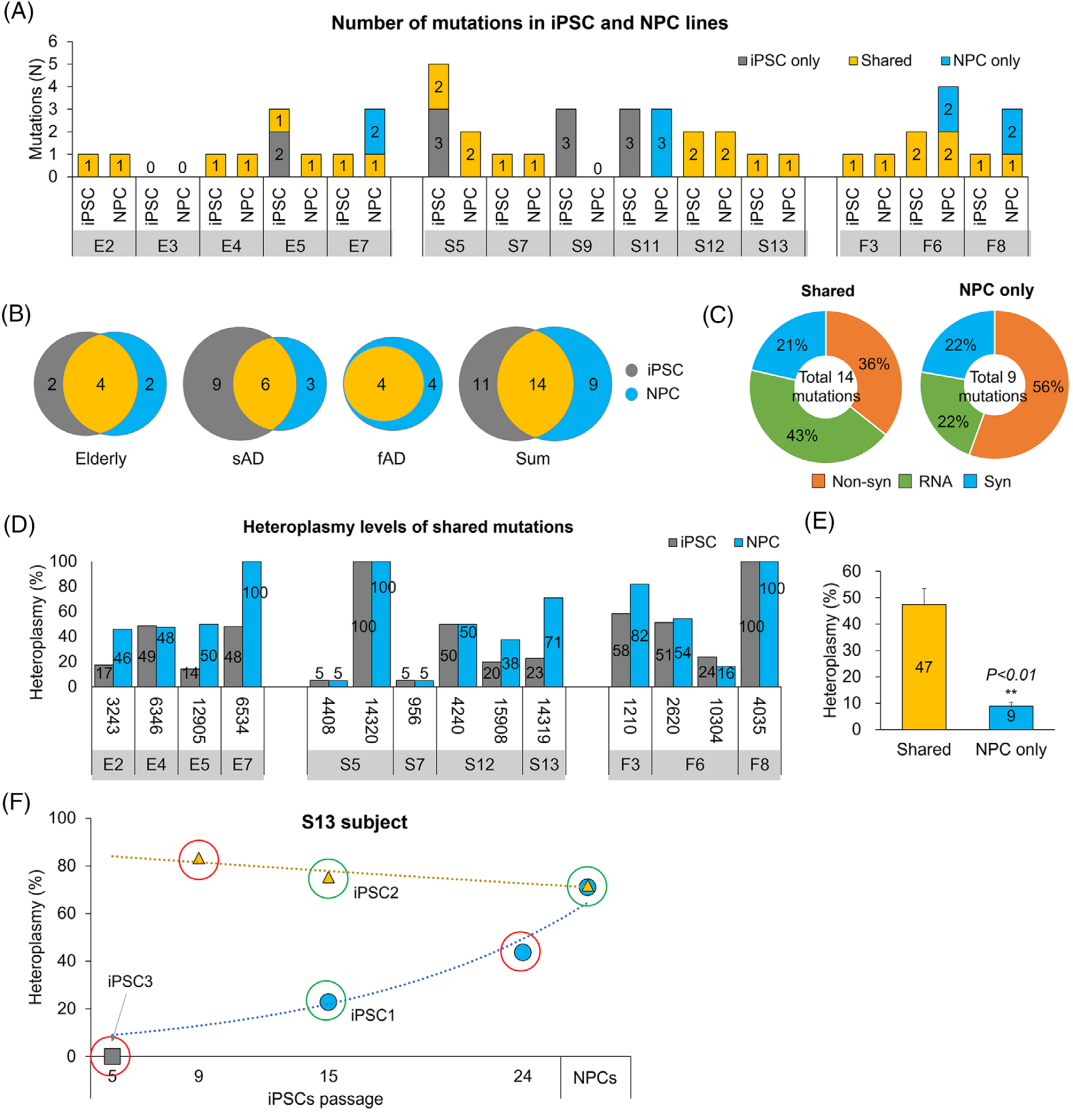
mtDNA mutations in neuronal precursor cells (NPCs) derived from iPSCs of elderly and subjects with AD. (A) The number of mtDNA mutations in each iPSC and iPSC‐derived NPCs. (B) The number of shared or unshared mtDNA mutations between iPSCs and NPCs in subject groups. Fourteen mutations were shared (four for elderly, six for sAD, and four for fAD subjects); other mutations detected only iPSC or NPC lines. (C) The distribution of mutation type for the shared mutations and the mutations detected only in NPCs. Nonsynonymous substitutions were 36% in the shared mutations, whereas 56% were detected in NPCs. Non‐syn, nonsynonymous; syn, synonymous; RNA, tRNA, or rRNA genes. (D) The heteroplasmy of shared mutations in iPSC and NPC lines. (E) The average heteroplasmy of the shared mutations and the mutations detected only in NPCs. Shared mtDNA mutations showed a significantly higher heteroplasmy than mutations detected only in NPCs. Mean ± SEM. **p < 0.01. (F) The heteroplasmic change of mt14319T>C mutation during expended iPSC culture and NPC differentiation in the S13 subject. iPSC1 showed a gradually increased heteroplasmy with extended culture and differentiation. However, iPSC2 displayed a similar heteroplasmy. iPSC3 had no mutation in mt14319. The cells with green circles were first analyzed for further analysis, followed by red circles. fAD, familial Alzheimer's disease; iPSC, induced pluripotent stem cell; sAD, sporadic Alzheimer's disease.

Next, we examined mtDNA mutations in NPCs derived from several iPSC lines of elderly and subjects with AD. Fourteen NPC lines and their original iPSC lines (five elderly, six sADs, and three fADs) were compared for the presence of mtDNA mutations. Six NPC lines and their original iPSC lines harbored only shared mutations (E2, E4, S7, S12, S13, and F3), whereas other iPSC and NPC lines contained some unshared mutations, detected only in iPSC or NPC lines (E5, E7, S5, S9, S11, F6, and F8) (Figure 3A). Among 34 mtDNA mutations, 14 mutations were shared between iPSCs and NPCs (4 for elderly, 6 for sAD, and 4 for fAD subjects), then 11 and 9 mutations were detected only in iPSC (2 for elderly and 9 for sAD subjects) or NPC lines (2 for elderly, 3 for sAD, and 4 for fAD subjects), respectively (Figure 3B). Also, 36% were nonsynonymous substitutions, whereas 43% were present in tRNA or rRNA genes among the shared mtDNA mutations (Figure 3C). mtDNA mutations, detected only in NPCs, displayed different distribution than the shared mutation, with 56% nonsynonymous substitutions and 22% RNA genes (Figure 3C). Finally, we compared the heteroplasmy of the shared mtDNA mutations in iPSC and NPC lines. Eight mutations showed similar heteroplasmic levels (mt6346 in E4, mt4408, and mt14320 in S5; mt956 in S7, mt4240 in S12, and mt2620; mt10304 in F6, and mt4305 in F8), whereas the rest mutations displayed higher heteroplasmy in NPC lines (Figure 3D). Furthermore, the average heteroplasmy of these shared mtDNA mutations was significantly higher than the mutations detected only in NPCs (Figure 3E).

In a previous study, mtDNA mutations in cultured cells were maintained upon their extended culture and subsequent differentiation.^12^ However, some mtDNA mutations of iPSCs in this study were not detected in differentiated NPCs, whereas some were detected only in the differentiated NPCs but not in the original iPSCs. Although mutations were detected in iPSCs and NPCs, the heteroplasmy of some mutations was unstable during differentiation. Among the shared mutations, mt14319T>C mutation detected in the S13 subject is known to be associated with early‐onset Parkinson's disease (PD),^16^ showing the most different heteroplasmy between iPSCs and NPCs in AD subjects. Therefore, we focused on this mutation to analyze the heteroplasmic level in more iPSCs and NPCs derived from the S13 subject.

The heteroplasmy was different between iPSCs (iPSC1, p.15) and iPSC1‐derived NPCs (23% vs. 71%); therefore, we analyzed another iPSC line (iPSC2, p.15) and iPSC2‐derived NPCs, and the resulting heteroplasmy was similar, showing 75% in iPSC2 and 72% in iPSC2‐derived NPCs (Figure 3F, green circles). The phenomena were different between iPSC1 and iPSC2; therefore, we analyzed more iPSC samples with different passage numbers (Figure 3F, red circles). iPSC1 (p.24) showed 44% heteroplasmy, which gradually increased with extended culture and differentiation (24% in p.15 iPSCs, 44% in p.24 iPSCs, and 71% in NPC). iPSC2 (p.9) had 83% heteroplasmy, indicating a similar heteroplasmy during iPSC culture and NPC differentiation for this iPSC line (83% in p.9 iPSCs, 75% in p.15 iPSCs, and 72% in NPC). Another iPSC line (iPSC3, p.5) showed no mutation in mt14319.

Since the dynamics of heteroplasmy for mtDNA mutation during iPSC culture and NPC differentiation were different in each iPSC line, especially the heteroplasmy, which was changed during in vitro culture for specific cell lines, we thawed the frozen iPSC lines with an earlier passage to identify the cause of this phenomenon.

### 3.4 Stable heteroplasmy in iPSC subclones and their derivatives

We hypothesized two possible mechanisms for the unstable heteroplasmy during culture and differentiation: preferential replication of specific mtDNA copy, or the ability of certain mtDNA to provide a growth advantage to the cells. To investigate mtDNA replication during culture, we dissociated iPSC1 and iPSC3 (p.4) of the S13 subject into single cells, cultured them, and isolated individual colonies (subclones). The iPSC2 was not subcloned because the line showed similar heteroplasmy during culture and differentiation (Figure 3F).

**FIGURE 4:**
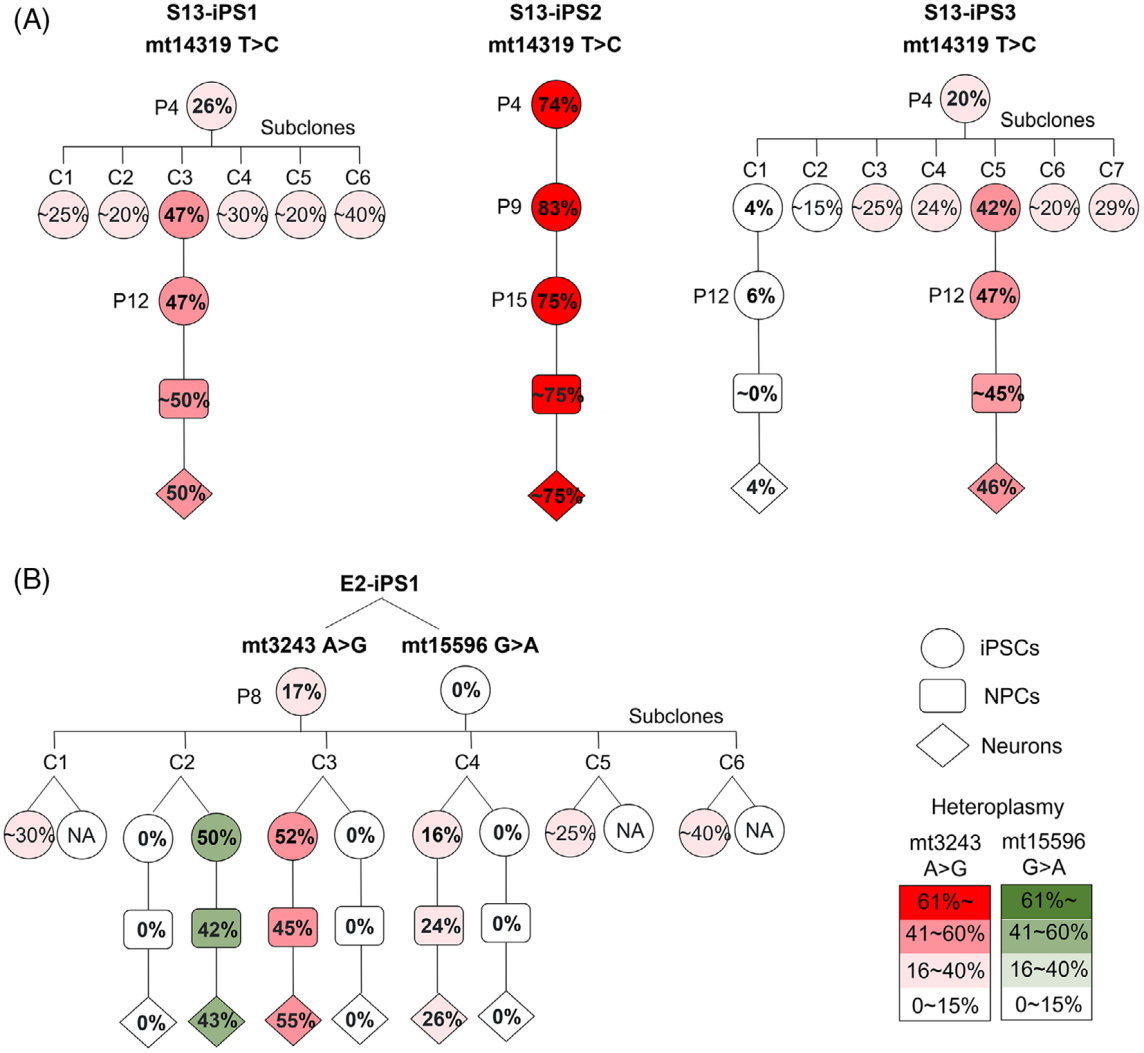
Stable heteroplasmy of iPSC subclones during extended culture and neuronal differentiation. (A) Stable heteroplasmy of mt14319T>C in iPSC subclones, NPCs, and neurons in S13 subject. The iPSC2 was not subcloned but showed a similar heteroplasmy during culture and differentiation. (B) Stable heteroplasmy of mtDNA mutations (mt3243A>G and mt15596G>A) in iPSC subclones, NPCs, and neurons in E2 subject. ~ indicates the quantified heteroplasmy by Sanger sequencing. iPSC, induced pluripotent stem cell; NPC, neuronal precursor cell

Six and seven subclones were established from iPSC1 and iPSC3, respectively, and their heteroplasmy of mt14319T>C mutation was analyzed (Figures 4A and S1A, and Table S5). Original iPSC1 and iPSC3 at passage 4 showed 26% and 20% heteroplasmy, respectively, whereas subclones displayed various heteroplasmy levels: 20%–47% for iPSC1 and 4%–42% for iPSC3. Furthermore, selected subclones of iPSC1 and iPSC3 (Clones 3 of iPSC1 and Clones 1 and 5 of iPSC3) and iPSC2 were analyzed for heteroplasmy during extended iPSC culture and neuronal differentiation, resulting that heteroplasmy level was similar in iPSCs that were passaged and differentiated into NPCs and neurons (Figures 4A and S1A).

To confirm the stable heteroplasmy of subclones, we generated subclones from iPSC of the elderly subject (E2 subject), which harbored mt3243A>G mutation, the most common mutation implicated in mitochondrial diabetes, mitochondrial encephalomyopathy, lactic acidosis, and stroke‐like episodes (MELAS).^17^ The original iPSC showed 17% heteroplasmy of mt3243A>G mutation, and six subclones displayed 0%–52% heteroplasmy (Figures 4B and S1B and Table S6). Subclones 2, 3, and 4 were differentiated into NPCs and neurons, and the heteroplasmy of subclones was also maintained during neuronal differentiation. One of the subclones included mt15596G>A mutation, which was not detected in the original iPSCs (Figures 4B and S1B and Table S6). This mutation also maintained the heteroplasmy during neuronal differentiation.

In summary, the mutations and their heteroplasmy levels of single‐cell clones were maintained during extended iPSC culture and neuronal differentiation, suggesting no preferential replication of mutant mtDNA within the cell during culture and differentiation.

### 3.5 Growth advantage of iPSCs carrying mtDNA mutations

**FIGURE 5:**
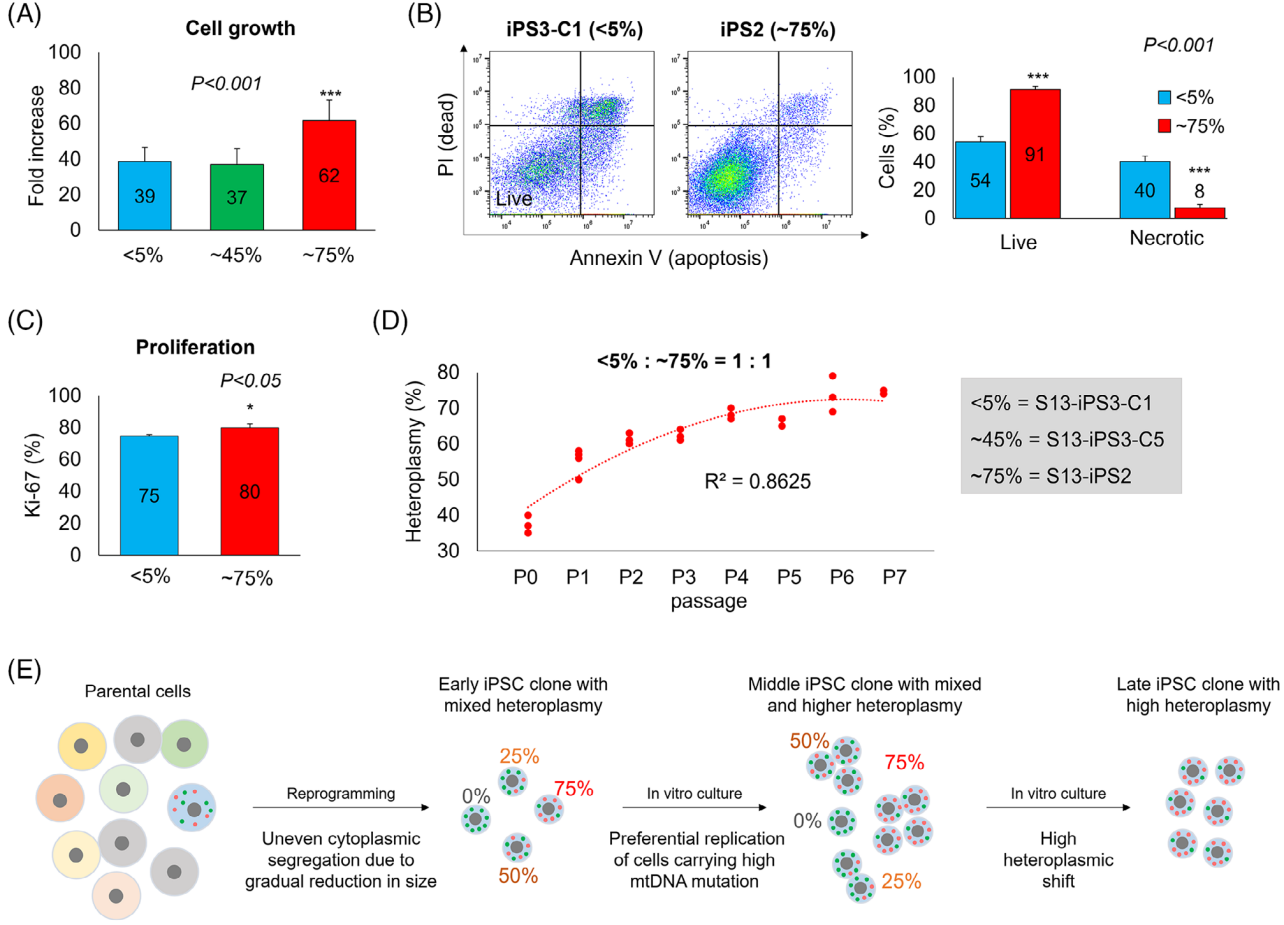
Growth advantage of iPSCs carrying mtDNA mutation. (A) Cell growth in iPSC subclones with different heteroplasmy of mt14319T>C, <5% in Clone 1 of iPSC3, ~45% in Clone 5 of iPSC3, and ~75% in iPSC2. Approximately 75% of clones showed a significantly higher fold increase than <5% and ~45% clones. ***p < 0.001. (B) The live and necrotic cells in <5% and ~75% clones. Approximately 75% of clones showed a significantly higher population for live cells and lower papulation for necrotic cells than <5% of clones. ***p < 0.001. (C) Cell proliferation in 4% and ~75% clone. Approximately 75% of clones showed significantly higher proliferation than <5% of clones. *p < 0.05. (D) The heteroplasmic change during passaging in mixed cell culture with <5% and ~75% clones. Heteroplasmy gradually increased with passaging, then reached around ~75% at passage 6. (E) Schematic hypothesis of how cells with mtDNA mutations could occupy whole cell populations during culture. Mean ± SEM. iPSC, induced pluripotent stem cell

We speculated that if cells harbor more mtDNA mutations, then they grow faster during culture. Cell growth is determined by cell viability during the splitting and proliferation of viable cells.^15^ Based on this hypothesis, we investigated the effect of mtDNA mutation and whether it can lead to a growth advantage for the cells. Three iPSC subclones with different heteroplasmy, Clone 1 of iPSC3 (<5%), Clone 5 of iPSC3 (~45%), and iPSC2 (~75%, p.15), were selected and their growth rates were compared. The fold increase in cell number was evaluated for cell growth. Approximately 75% of clones showed a significantly higher fold increase than <5% and ~45% clones (62‐fold vs. 39‐ and 37‐fold, Figure 5A). To understand the different growth mechanisms according to the level of heteroplasmy, we first compared live and apoptotic cell populations between <5% and ~75% clones 24 h after cell seeding. Approximately 75% clones showed significantly higher live cell population and lower necrotic cell population than <5% clone (91% vs. 54% for live‐cell population; 8% vs. 40% for necrotic cell population) (Figure 5B). Ki‐67 protein expression was also examined to investigate cell proliferation, which showed that ~75% of clones showed a significantly higher proliferation than <5% (Figure 5C). To ascertain that mtDNA mutation provides the advantage of cell growth, <5% and ~75% clones were mixed in a 1:1 ratio and cultured to passage 7. The initial mtDNA mutation level was ~35% and was gradually increased with passaging, then reached ~75% at passage 6, and this level was maintained at passage 7, suggesting ~75% clone exhibited a faster cell growth rate than <5% one (Figure 5D). The iPSCs with higher mtDNA mutations represented a growth advantage due to the high viability and proliferation of cells.

Based on these results, we hypothesized how cells with higher mtDNA mutations eventually occupied the whole population. During the iPSC reprogramming, the size of cells could gradually reduce, resulting in uneven segregation of the cytoplasm (Figure 5E).^18^ Therefore, the iPSCs with early passage contained various levels of mtDNA mutation (Figure 5E). The cells with mtDNA mutation could have preferential replication during iPSC culture, resulting in a high heteroplasmic shift in the late passage. The phenomenon would appear among different mtDNA mutations. If one mutation could induce faster growth in cells than in others, cells carrying that mutation can take over the entire iPSCs.

### 3.6 Low mitochondrial function in neuronal lineage with mtDNA mutations

To examine whether mtDNA mutation could affect mitochondrial function, three iPSC clones with different heteroplasmy; Clone 1 of iPSC3 (<5%), Clone 5 of iPSC3 (~45%), and iPSC2 (~75%), were selected, differentiated into the neuronal lineage, and their mitochondrial function was evaluated.

**FIGURE 6:**
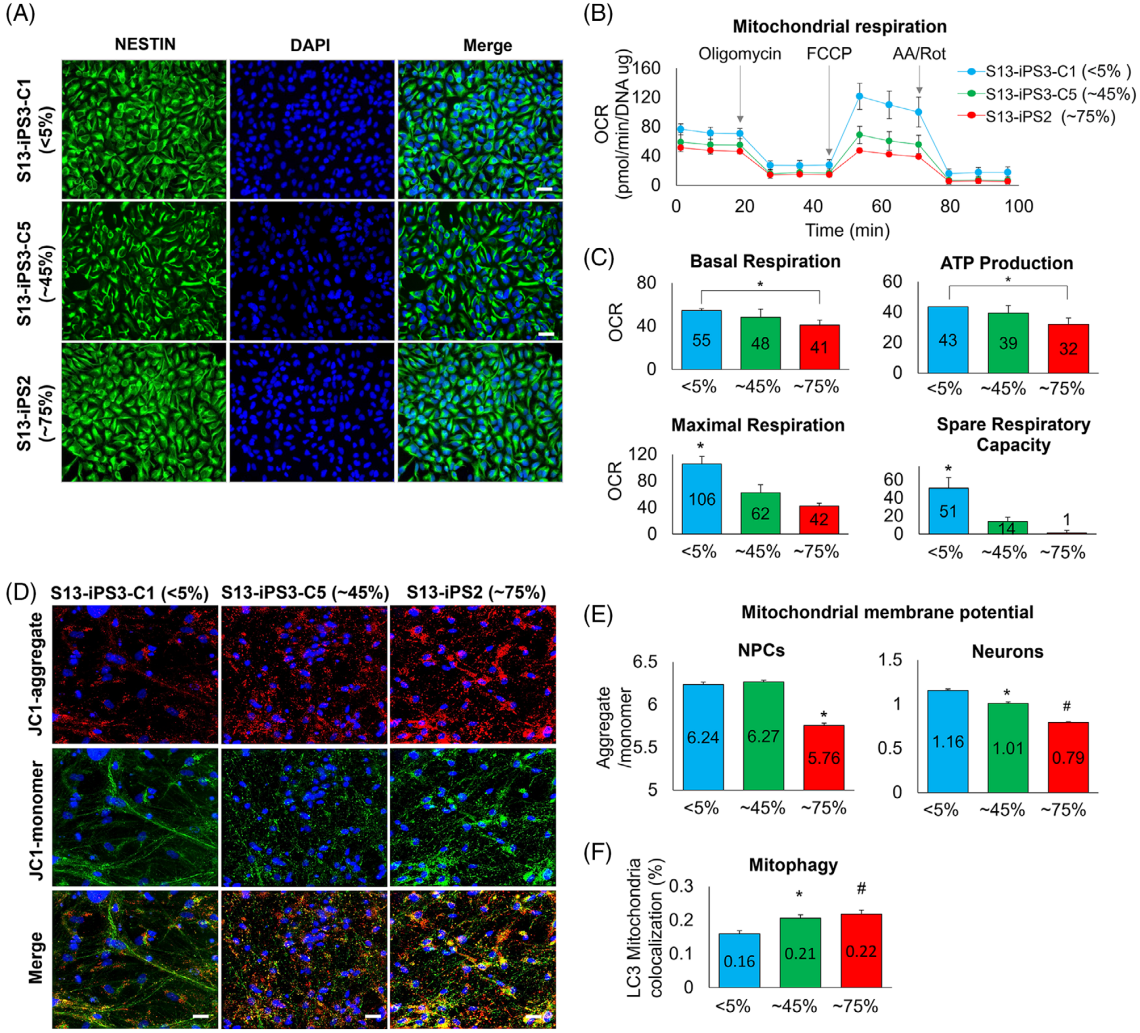
Mitochondrial quality defects due to mutated mtDNA. (A) The expression of a lineage‐specific marker, NESTIN, in Clone 1 of iPSC3 (<5% heteroplasmy of mt14319T>C), Clone 5 of iPSC3 (~45%), and iPSC2 (~75%). All NPC lines expressed NESTIN. Scale bar = 20 μm. (B and C) Mitochondrial respiration in NPCs. NPCs differentiated from ~75% iPSC clones exhibited a lower OCR rate than that derived from <5% iPSC clones. (D) Expression of JC‐1 monomers and JC‐1 aggregates in neurons. JC‐1 monomers form JC‐1 aggregates in healthy mitochondria. Scale bar = 20 μm. (F) Mitochondrial membrane potential (MMP) in NPCs and neurons. MMP was significantly lower in NPCs and neurons from ~75% iPSC clones than those from <5% and ~45% clones. (F) Mitophagy in neurons. Neurons from ~75% of clones showed increased mitophagy than those from <5% and ~45% clones. * and ^#^ *p* < 0.05. mean ± SD for panel B, mean ± SEM for panels C, E, and F. iPSC, induced pluripotent stem cell; NPC, neuronal precursor cell

First, iPSC clones were differentiated into NPCs, and their lineage‐specific marker, NESTIN, showed positive expression in all NPC lines (Figure 6A). Next, we measured mitochondrial OCR to evaluate the mitochondrial respiratory function for NPC lines.^12^ NPCs differentiated from <5% iPSC clones exhibited a higher OCR rate compared with those from ~45% and ~75% clones (Figure 6B). NPCs from ~75% clone showed significantly lower basal respiration, ATP production, maximal respiration, and spare respiratory capacity than those from <5% clone (Figure 6C). NPCs from ~45% clone displayed significantly lower OCR in maximal respiration and spare respiratory capacity than those from <5% clone. These findings demonstrated that high heteroplasmic mtDNA mutation could affect mitochondrial respiratory function in differentiated NPCs.

Next, mitochondrial membrane potential (MMP), a key indicator of mitochondrial viability and homeostasis,^19^ was also measured in differentiated NPCs and neurons derived from iPSC clones with <5%, ~45%, and ~75% heteroplasmy. JC‐1 dye was used for measuring MMP, of which JC‐1 monomers form JC‐1 aggregates in healthy mitochondria (Figure 6D and S2A).^20^ The MMP level was significantly lower in NPCs and neurons derived from ~75% iPSC clones than those from <5% and ~45% clones (Figure 6E). Neurons from ~45% clones also showed a significantly lower MMP than that from <5% clones. Finally, we assessed the ability of mitophagy to selectively degrade mitochondria by autophagy, an important mechanism for mitochondrial quality control by eliminating damaged mitochondria.^21^ We observed that neurons from ~75% clones showed significantly increased mitophagy than those from <5% and ~44% clones (Figure 6F and S2B).

In summary, the high heteroplasmic mtDNA mutations resulted in diminished mitochondrial respiratory function in differentiated NPCs. Furthermore, reduced MMP and increased mitophagy were observed in differentiated NPCs or neurons with high heteroplasmy. These results suggested that mtDNA mutations could induce mitochondrial dysfunction.

### 3.7 Occurrence of higher AD phenotypes in neuronal cells differentiated from iPSCs carrying mtDNA mutations

A previous report showed that mtDNA integrity affects neuronal differentiation fate, such as the lack of neurogenesis and elevated astrogliosis, in *Ogg1* deficient transgenic mice.^22^ To determine whether mtDNA mutation could interfere with neuronal differentiation in iPSCs of patients with AD, three iPSC clones with <5% (Clone 1 of iPSC3), ~45% (Clone 5 of iPSC3), and ~75% (iPSC2) heteroplasmy were differentiated into neurons and the composition of neurons and astrocytes, and their outgrowth and pathogenesis were investigated.

**FIGURE 7:**
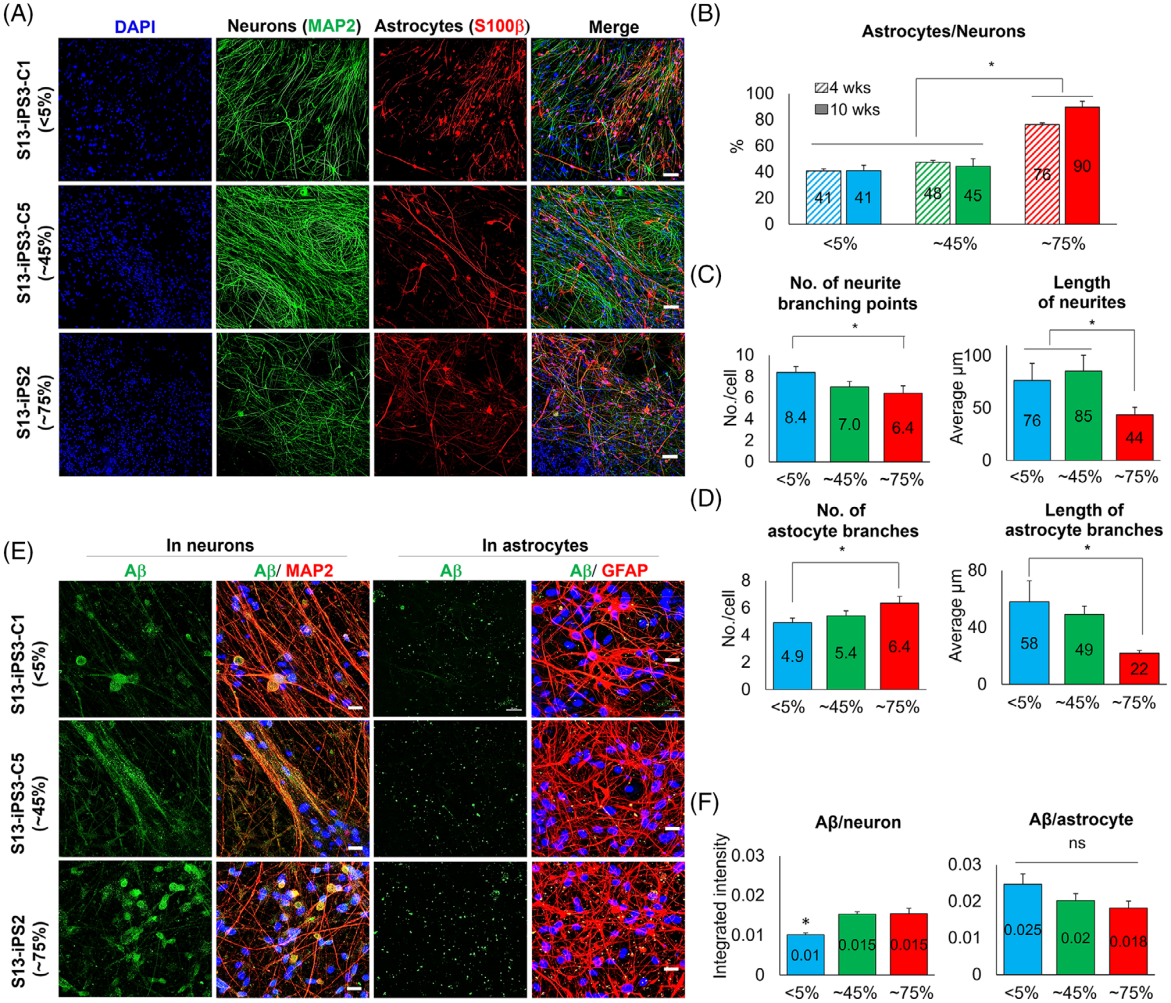
Impaired neuronal differentiation due to mtDNA mutation. (A) MAP2 expression in neurons and S100β expression in astrocytes in Clone 1 of iPSC3 (<5% heteroplasmy of mt14319T>C), Clone 5 of iPSC3 (~45%), and iPSC2 (~75%). Scale bar = 100 μm. (B) The population ratio between neurons and astrocytes after neuronal differentiation depended on the heteroplasmy of mtDNA mutation. Astrogliosis was significantly higher in ~75% of clones than in <5% and ~45% clones at 4 and 10 weeks of differentiation. (C) The number of branching points and length of neurites in differentiated neurons. Neurons from ~75% clone showed a significantly lower number of neurite branching points and shorter length of neurites than those from <5% and ~45% clone, respectively. (D) The number and length of astrocyte branches after differentiation. The astrocytes from ~75% clones showed significantly more branches but a shorter length than that of <5% clone. (E and F) Depositions of β‐amyloid (Aβ) in differentiated neurons and astrocytes. Aβ deposition was significantly higher in neurons from ~45% and ~75% clone than those from 4% clone. However, there was no significant difference in differentiated astrocytes. Scale bar = 20 μm. ns, not significant. Mean ± SEM. *p < 0.05

After iPSC clones were differentiated into neurons for 4 and 10 weeks, MAP2 expression in neurons and S100β expression in astrocytes were measured to evaluate the population ratio between neurons and astrocytes (Figure 7A).^23^, ^24^ Astrogliosis was significantly higher in ~75% iPSC clone than in <5% and ~45% clone at 4 and 10 weeks of differentiation (Figure 7B). However, the duration of differentiation did not affect the ratio of astrogliosis in all iPSC clones (Figure 7B).

We analyzed the number and length of neurites in neurons and branches of astrocytes. Based on the similar number of neurites among the neurons from <5%, ~45%, and ~75% clones, the number of neurite branching points was analyzed, resulting in a significantly lower number of neurons from ~75% clone than that of <5% clone (Figure 7C). Also, the average length of neurites was significantly shorter in neurons from ~75% clone than that of ~45% clone. The ~75% clone astrocytes showed significantly more branches but had a shorter average length than that of <5% clone (Figure 7D).

The deposition of Aβ, the key component in the pathogenesis of AD,^25^ was analyzed in MAP2‐positive neurons and GFAP‐positive astrocytes (Figure 7E). The Aβ accumulation was significantly higher in neurons derived from ~45% and ~75% clones than those from <5% clones (Figure 7F). However, Aβ accumulation in astrocytes was decreased with higher heteroplasmy, but no significant difference was observed.

Based on these results, we demonstrated that iPSC clones with a high heteroplasmy showed deficient neurogenesis, including shorter length and fewer branching points of neurites, and elevated Aβ accumulation. Differentiated astrocytes from high heteroplasmic iPSC clones also showed short branches, but there was no significant difference in Aβ accumulation compared with low heteroplasmic clones. Thus, we concluded that mtDNA mutation could inhibit neuronal differentiation and promote pathogenesis in differentiated neurons.

## 4 DISCUSSION

Mitochondria are essential intracellular organelles responsible for energy production, calcium signaling, cell apoptosis, and death.^26^, ^27^ In neurons, mitochondria are critical in neuronal survival, neurotransmission, and plasticity, whereas mitochondria‐mediated defects could lead to the pathogenesis of neurological diseases such as AD, PD, and Huntington's disease.^28^, ^29^ In this study, we investigated the influence of mtDNA mutations on neuronal differentiation in patients with AD, demonstrating that the mtDNA mutations could induce mitochondrial dysfunction and pathogenesis in differentiated neuronal cells.

We demonstrated that mtDNA mutation could increase cell growth due to its high viability and proliferation. When the iPSC clones with different heteroplasmic mtDNA mutations were mixed and cultured, the heteroplasmy of mtDNA mutations increased during cell culture, caused by the growth advantage of the iPSC clone with high heteroplasmic mtDNA mutations. This phenomenon was also observed in pooled iPSCs before subcloning. Furthermore, this growth advantage led to the same mutations in multiple cells and increased heteroplasmy during the cell culture in pooled iPSCs. Based on this result, we suggested that pathogenic mutations could be distributed at high rates in neurons in the brain due to the growth advantage, which may affect the pathogenicity of neuronal diseases such as AD.

We selected iPSC clones harboring mutations with different heteroplasmy to evaluate that mtDNA mutation could affect neuronal differentiation. The mtDNA mutation in the *ATP6* gene induces defective ATP production, abnormal MMP, and altered calcium homeostasis in differentiated NPC.^30^ The mtDNA mutation in the current study was located in the *ND6* gene. A higher heteroplasmy level of this mutation resulted in diminished mitochondrial respiratory function and a reduced MMP, suggesting that this mutation could also play a critical role in mitochondrial dysfunction in patients with AD. Furthermore, mitophagy was increased in iPSC clones with high heteroplasmic mtDNA mutation, possibly correlated to the elimination of dysfunctional mitochondria.^31^ mtDNA mutations could alter cell signaling pathways, leading to mitochondrial quality control processes such as mitophagy.^32^ Previous studies demonstrated that mitophagy is impaired in neurodegenerative disorders such as AD.^21^, ^33^ However, the current study showed increased mitophagy in differentiated neurons with elevated deposition of Aβ. We suggested that Aβ deposition by mtDNA mutation could represent the different phenomena of mitophagy, needing further studies involving mtDNA mutations and mitophagy in AD.

A previous study demonstrated that mtDNA integrity could affect neuronal differentiation, and mtDNA damage can induce decreased neurogenesis and increased astrogliosis during the repair of neuronal injury.^22^ Our results showed a similar phenomenon, where iPSC clones with high heteroplasmic mutation exhibited a high ratio of astrocytes after neuronal differentiation. Furthermore, differentiated neurons with high heteroplasmic mutation displayed deficient neurite outgrowth, which has also been reported in other neuronal diseases such as MELAS, and myoclonic epilepsy with ragged‐red fibers syndrome.^34^, ^35^

For Aβ production, there was a question of whether neurons differentiated from cells other than brain‐associated cells, such as blood cells, could deposit Aβ. A previous study demonstrated that the neuronal cells, differentiated from MNCs‐ or dermal fibroblast‐derived iPSCs, were measured for Aβ production without any gene editing or chemical treatment.^36^, ^37^ Based on this report, we hypothesized the possibility of Aβ accumulation in neurons differentiated from MNC‐derived iPSCs in patients with AD, which showed that differentiated neurons deposited Aβ. We used three iPSC clones with different heteroplasmic mtDNA mutations (<5%, ~45%, and ~75%) and all clones were shown to produce Aβ. In addition, the deposition of Aβ was higher in iPSC clones with high heteroplasmic mtDNA mutation, suggesting that mtDNA mutation could accelerate Aβ accumulation in differentiated neurons.

In summary, our research revealed that mtDNA mutations, known to induce cell growth advantage, could lead to mitochondrial dysfunction and Aβ accumulation in differentiated neurons in patients with AD. Therefore, it is essential to screen for mtDNA mutations and select mutation‐free iPSC lines before using them to differentiate into specific cell types for clinical application.

## AUTHOR CONTRIBUTIONS

*Study design*: Jihwan Song and Eunju Kang. *Data collection and analysis*: Yeonmi Lee, Minchul Kim, Miju Lee, Seongjun So, Soon‐Suk Kang, Jiwan Choi, Deokhoon Kim, Hee Ra Park, and Sung Soo Lee. *Manuscript preparation*: Yeonmi Lee, Minchul Kim, Jihwan Song, and Eunju Kang. *Financial support and manuscript revision*: Jihwan Song, Eunju Kang, and Jung Jae Ko.

## CONFLICT OF INTEREST

Jihwan Song is the founder and CEO of iPS Bio, Inc. The other authors declare no conflict of interest.

## Supporting information


**Appendix S1** Supporting information

**TABLE S3** mtDNA mutations in UCB subjects (Excel file).

**TABLE S4** mtDNA mutations in elderly subjects (Excel file).

**TABLE S5** mtDNA mutations in the subjects with sporadic Alzheimer's disease (Excel file).

**TABLE S6** mtDNA mutations in the subjects with familial Alzheimer's disease (Excel files).

## Data Availability

The data that supported the findings of this study are available on request from the corresponding authors.
