# Supplementary material for: Mitochondrial genome mutations and neuronal dysfunction of induced pluripotent stem cells derived from patients with Alzheimer's disease
Source: Cell Prolif. 2022 Jun 13;55(7):e13274. doi: 10.1111/cpr.13274 (PMC9251050; doi:10.1111/cpr.13274)
Supplement: Supplementary file 1 — Appendix S1 Supporting information [file CPR-55-e13274-s004.docx]

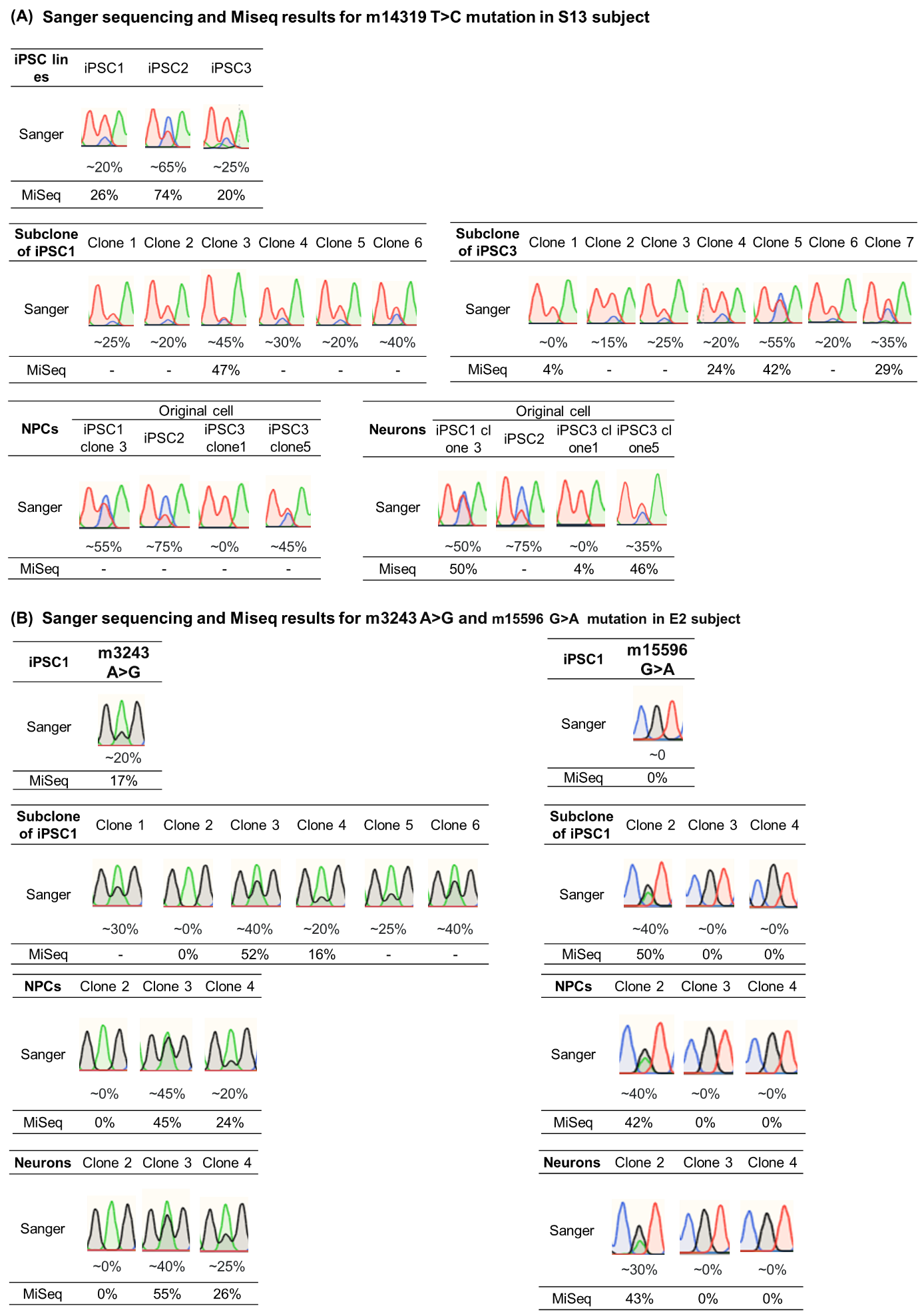


**FIGURE S1.** Sanger sequencings and corresponding Miseq results of iPSC subclones, differentiated NPCs and neurons in S13 and E2 subject. (A) Sanger sequencing and corresponding Miseq results for m14319 T>C mutation in S13 subject. (B) Sanger sequencing and corresponding Miseq results for m3243 A>G and m15596 G>A mutations in E2 subject. ~ indicates the quantified heteroplasmy by Sanger sequencing.


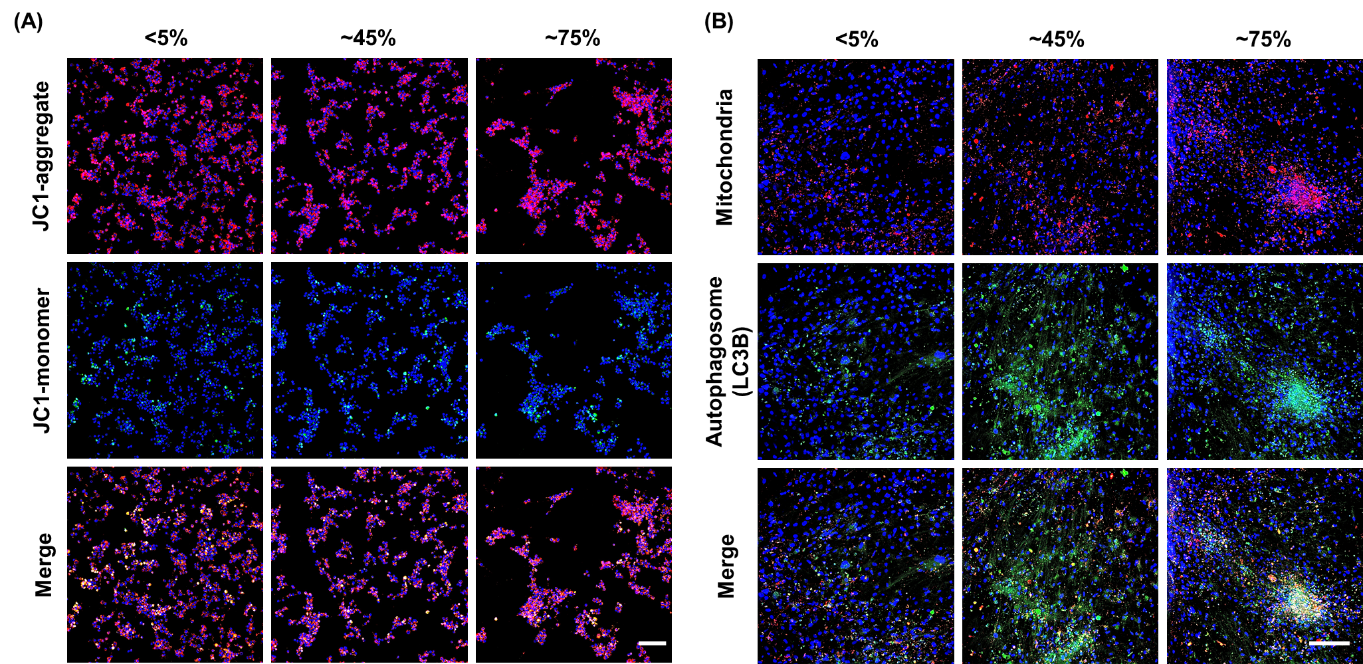


**FIGURE S2.** Mitochondrial membrane potential in NPCs and mitophagy in neurons derived from in clone 1 of iPSC3 (<5% heteroplasmy of mt14319 T>C), clone 5 of iPSC3 (~45%), and iPSC2 (~75%). (A) The expression of JC-1 monomers and JC-1 aggregates in NPCs. (B) The expression of human mitochondria and LC3B, an autophagosome marker, in neurons. Scale bar = 100 μm.

**TABLE S1**. List of subjects

| Group | **Subject code** | **Sex** | **Age (Age)** | **mtDNA**  **haplogroup** | **No. of**  **iPSC lines** |
| --- | --- | --- | --- | --- | --- |
| UCB | U1 | N/A | 0 | D | 1 |
|  | U2 | N/A | 0 | D | 1 |
|  | U3 | N/A | 0 | M | 1 |
|  | U4 | N/A | 0 | D | 1 |
|  | U5 | N/A | 0 | D | 1 |
|  | U6 | N/A | 0 | B | 1 |
|  | U7 | N/A | 0 | M | 1 |
|  | U8 | N/A | 0 | D | 1 |
|  | U9 | N/A | 0 | F | 1 |
|  | U10 | N/A | 0 | A | 1 |
| Elderly | E1 | F | 55 | D | 2 |
|  | E2 | M | 60 | B | 2 |
|  | E3 | F | 61 | M | 2 |
|  | E4 | M | 72 | D | 2 |
|  | E5 | M | 80 | A | 2 |
|  | E6 | F | 81 | N | 1 |
|  | E7 | F | 84 | N | 1 |
| Sporadic Alzheimer’s disease | S1 | F | 53 | N | 2 |
|  | S2 | M | 56 | D | 1 |
|  | S3 | F | 58 | A | 1 |
|  | S4 | F | 59 | D | 1 |
|  | S5 | M | 62 | A | 1 |
|  | S6 | F | 73 | D | 2 |
|  | S7 | F | 73 | N | 2 |
|  | S8 | F | 76 | C | 1 |
|  | S9 | M | 77 | A | 2 |
|  | S10 | M | 79 | F | 1 |
|  | S11 | M | 79 | Y | 2 |
|  | S12 | F | 83 | D | 1 |
|  | S13 | M | 86 | M | 3 |
| Familial Alzheimer’s disease | F1 | F | 24 | D | 2 |
|  | F2 | M | 32 | C | 1 |
|  | F3 | M | 34 | D | 2 |
|  | F4 | M | 35 | A | 1 |
|  | F5 | F | 40 | B | 1 |
|  | F6 | F | 45 | F | 1 |
|  | F7 | F | 53 | D | 1 |
|  | F8 | M | 54 | M | 2 |
|  | F9 | M | 59 | M | 2 |
|  | F10 | F | 63 | D | 2 |
| Total | 40 |  |  |  | 59 |

N/A means not applicable.

**TABLE S2.** Summary of mtDNA mutations in iPSC lines and differentiated NPCs

| Group | Subject | Position | iPSC1 | iPSC2 | NPC | | Effect |
| --- | --- | --- | --- | --- | --- | --- | --- |
|  |  |  | Heteroplasmy (%) | | Original cell | Heteroplasmy (%) |  |
| UCB | U5 | 6018 | 15.3 | N/A | N/A | | COI:A=>T |
| Elderly | E1 | 875 | 91.4 | 86.36 | N/A | | rRNA |
|  |  | 4891 | 54.2 | 54.17 |  |  | ND2:I=>T |
|  |  | 14985 | 25.8 | 18.28 |  |  | Cytb:R=>H |
|  | E2 | 3243 | 17.32 | 16.67 | iPSC1 | 45.8 | tRNA |
|  | E4 | 6346 | 48.72 | 48.72 | iPSC1 | 47.5 | COI:F=>S |
|  |  | 11176 | 0 | 7.07 |  | 0 | ND4:Q=>Q |
|  |  | 15757 | 0 | 4.92 |  | 0 | Cytb:W=>C |
|  | E5 | 2721 | 17.72 | 0 | iPSC2 | 0 | rRNA |
|  |  | 4136 | 4.95 | 13.76 |  | 0 | ND1:Y=>C |
|  |  | 12905 | 0 | 14.12 |  | 50 | ND5:I=>T |
|  |  | 13874 | 0 | 10.58 |  | 0 | ND5:M=>T |
|  | E6 | 5258 | 27.5 | N/A | N/A | | ND2:K=>K |
|  | E7 | 2487 | 0 | N/A | iPSC1 | 5.6 | rRNA |
|  |  | 6534 | 47.8 |  |  | 100 | COI:T=>A |
|  |  | 9723 | 0 |  |  | 16.9 | COIII:F=>L |
| Sporadic Alzheimer’s disease | S1 | 4011 | 0 | 4.8 | N/A | | ND1:N=>N |
|  |  | 5258 | 0 | 45.5 |  |  | ND2:K=>K |
|  |  | 10700 | 48.4 | 0 |  |  | ND4L:L=>L |
|  | S3 | 2063 | 5.1 | N/A | N/A | | rRNA |
|  |  | 12860 | 12.3 |  |  |  | ND5:N=>S |
|  |  | 15295 | 5.6 |  |  |  | Cytb:F=>F |
|  | S4 | 1169 | 5.8 | N/A | N/A | | rRNA |
|  |  | 12952 | 100 |  |  |  | ND5:A=>S |
|  | S5 | 1623 | 5.5 | N/A | iPSC1 | 0 | tRNA |
|  |  | 4408 | 5.0 |  |  | 5.0 | tRNA |
|  |  | 5628 | 7.1 |  |  | 0 | tRNA |
|  |  | 13879 | 4.0 |  |  | 0 | ND5:S=>P |
|  |  | 14320 | 100 |  |  | 100 | ND6:F=>F |
|  | S6 | 2998 | 13.9 | 0 | N/A | | rRNA |
|  |  | 11914 | 48.4 | 0 |  |  | ND4:T=>T |
|  | S7 | 956 | 5.0 | 5.4 | iPSC1 | 4.9 | rRNA |
|  | S8 | 1313 | 5.8 | N/A | N/A | | rRNA |
|  |  | 3709 | 8.3 | N/A |  |  | ND1:A=>S |
|  | S9 | 1068 | 29.3 | 0 | iPSC1 | 0 | rRNA |
|  |  | 1189 | 19.3 | 3.9 |  | 0 | rRNA |
|  |  | 1661 | 12.6 | 0 |  | 0 | tRNA |
|  | S10 | 9315 | 14.0 | N/A | N/A | | COIII:F=>L |
|  | S11 | 4597 | 6.7 | 0.0 | iPSC1 | 0 | ND2:V=>A |
|  |  | 4772 | 0.0 | 0.0 |  | 7.7 | ND2:A=>A |
|  |  | 6070 | 9.5 | 9.4 |  | 0 | COI:V=>A |
|  |  | 6905 | 5.5 | 0.0 |  | 0 | COI:W=>W |
|  |  | 7342 | 0 | 0 |  | 14.2 | COI:R=>Q |
|  |  | 15270 | 0 | 0 |  | 3.6 | Cytb:L=>P |
|  | S12 | 4240 | 50.0 | N/A | iPSC1 | 50.0 | ND1:S=>P |
|  |  | 15908 | 19.7 | N/A |  | 37.5 | tRNA |
|  | S13 | 14319 | 22.7 | 75.3 | iPSC1 | 71.0 | ND6:N=>D |
|  |  | 11563 | 0 | 9.0 |  | 0 | ND4:G268G |
| Familial Alzheimer’s disease | F1 | 1345 | 16.5 | 0 | N/A | | rRNA |
|  |  | 2608 | 0 | 8.2 |  |  | rRNA |
|  |  | 4048 | 0 | 4.7 |  |  | ND1:D=>N |
|  |  | 11432 | 5.3 | 0 |  |  | ND4:I=>V |
|  |  | 13327 | 8.0 | 0 |  |  | ND5:T=>A |
|  | F2 | 10943 | 100 | N/A | N/A | | ND4:T=>S |
|  |  | 13369 | 16.2 |  |  |  | ND5:S=>P |
|  |  | 15172 | 42.9 |  |  |  | Cytb:G=>G |
|  | F3 | 1210 | 22.7 | 58.3 | iPSC2 | 81.8 | rRNA |
|  | F4 | 9522 | 100 | N/A | N/A | | COIII:L=>L |
|  |  | 12905 | 10.4 |  |  |  | ND5:I=>T |
|  | F5 | 8784 | 98.2 | N/A | N/A | | ATPase6:G=>G |
|  | F6 | 2620 | 51.2 | N/A | iPSC1 | 54.4 | rRNA |
|  |  | 4239 | 0 |  |  | 6.4 | ND1:I=>I |
|  |  | 10304 | 23.8 |  |  | 16.1 | ND3:T=>T |
|  |  | 14268 | 0 |  |  | 9.3 | ND6:R=>TERM |
|  | F7 | 2620 | 51.2 | N/A | N/A | | rRNA |
|  |  | 8831 | 4.8 | N/A |  |  | ATPase6:L=>P |
|  |  | 13633 | 5.4 | N/A |  |  | ND5:G=>S |
|  |  | 15975 | 3.5 | N/A |  |  | tRNA |
|  | F8 | 2806 | 0 | 0 | iPSC1 | 3.9 | rRNA |
|  |  | 4035 | 100 | 100 |  | 100 | ND1:L=>L |
|  |  | 11601 | 0 | 0 |  | 11.8 | ND4:D=>A |
|  | F9 | 1887 | 50.0 | 0 | N/A | | rRNA |
|  |  | 2626 | 4.4 | 0 |  |  | rRNA |
|  |  | 7809 | 3.9 | 0 |  |  | COII:L=>P |
|  |  | 9161 | 0 | 14.8 |  |  | ATPase6:Y=>C |
|  |  | 12668 | 5.5 | 0 |  |  | ND5:D=>G |
|  | F10 | 1336 | 0 | 8.3 | N/A | | rRNA |
|  |  | 8697 | 14.5 | 11.5 |  |  | ATPase6:M=>M |
|  |  | 9307 | 5.7 | 6.6 |  |  | COIII:W=>TERM |
|  |  | 15479 | 87.2 | 88.3 |  |  | Cytb:F=>L |

N/A means not applicable.

**TABLE S3.** mtDNA mutations in UCB subjects (Excel file).

**TABLE S4.** mtDNA mutations in elderly subjects (Excel file).

**TABLE S5.** mtDNA mutations in the subjects with sporadic Alzheimer’s disease (Excel file).

**TABLE S6.** mtDNA mutations in the subjects with familial Alzheimer’s disease (Excel files).
